# Little evidence that posttraumatic stress is associated with diurnal hormone dysregulation in Turkana pastoralists

**DOI:** 10.1093/emph/eoaf004

**Published:** 2025-02-17

**Authors:** Matthew R Zefferman, Michael D Baumgarten, Benjamin C Trumble, Sarah Mathew

**Affiliations:** Department of Defense Analysis, Naval Postgraduate School, Monterey, CA, 93943, USA; Institute of Human Origins, Arizona State University, Tempe, AZ, 85281, USA; Institute of Human Origins, Arizona State University, Tempe, AZ, 85281, USA; School of Human Evolution and Social Change, Arizona State University, Tempe, AZ, 85281, USA; Institute of Human Origins, Arizona State University, Tempe, AZ, 85281, USA; School of Human Evolution and Social Change, Arizona State University, Tempe, AZ, 85281, USA; Center for Evolution and Medicine, Arizona State University, Tempe, AZ, 85281, USA; Institute of Human Origins, Arizona State University, Tempe, AZ, 85281, USA; School of Human Evolution and Social Change, Arizona State University, Tempe, AZ, 85281, USA

**Keywords:** PTSD, combat stress, cortisol, testosterone, posttraumatic, pastoralists, cattle raids

## Abstract

Research in industrialized populations suggests that posttraumatic stress disorder (PTSD) may be associated with decreased cortisol or testosterone sensitivity, resulting in a blunted diurnal rhythm. However, the evolutionary implications of this association are unclear. Studies have primarily been conducted in Western industrialized populations, so we do not know whether hormonal blunting is a reliable physiological response to PTSD or stems from factors unique to industrialized settings. Furthermore, existing studies combine PTSD from diverse types of traumas, and comparison groups with and without PTSD differ along multiple dimensions, making it hard to know if PTSD or other life factors drive the blunted cortisol response. We conducted a study among *n* = 60 male Turkana pastoralists, aged between about 18–65 years in Kenya, exposed to high levels of lethal inter-ethnic cattle raiding. 28% of men in this area have PTSD symptom severity that would qualify them for a provisional PTSD diagnosis. Saliva samples were collected at three points to compare the cortisol and testosterone profiles of Turkana warriors with and without PTSD. Contrary to existing work, our preregistered analysis found little evidence for a difference in the hormonal profiles of warriors with high versus low PTSD symptom severity. Our results imply that the relationship between PTSD and hormonal diurnal variation may vary across populations and ecologies or that the association documented in Western populations stems from other correlated life factors. Studies in a wider range of populations and ecological contexts are needed to understand the evolutionary underpinnings of hormonal responses to trauma.

## BACKGROUND AND OBJECTIVES

Posttraumatic stress disorder (PTSD) is a mental health disorder that can develop after an individual is exposed to ‘actual or threatened death, serious injury, or sexual violence’ [1]. While approximately 80% of the US population will experience a traumatic experience at some point in their life, approximately 3.4%–8.0% of civilians will be diagnosed with PTSD [2]. The prevalence in members of American operational infantry units deployed to Iraq and Afghanistan is estimated to be around 13% [3], with lifetime prevalence for all veterans ranging from 7.7% to 13.4% [2].

Some literature suggests that PTSD is an evolutionarily deep-rooted mammalian response to acute life-threatening events that enables individuals to learn from and avoid physical dangers [4–7]. Based on evidence from Turkana pastoralists with combat exposure, Zefferman and Mathew proposed at least two evolutionary origins for PTSD symptoms [8]. The first includes symptoms that may have deep evolutionary roots and be associated with learning from and reacting to the dangerous event—memories, nightmares, flashbacks, cued distress, cued physical reactions, enhanced startle, and hypervigilance. These symptoms occurred at similar frequencies in high-severity Turkana warriors and American service members and were particularly associated with combat exposure in the Turkana [8].

On the other hand, Zefferman and Mathew argued that PTSD symptoms resembling depression, sometimes categorized as ‘dysphoric symptoms’ [9]—low concentration, detachment, irritability, loss of interest, negative beliefs, negative feelings, and numbing—may be an evolutionarily recent response specific to humans that enables individuals to respond to morally risky situations by modulating their motivation to engage in actions that might be socially sanctioned and expressing remorse if they have violated communal norms [8]. This is similar to some evolutionary explanations for depression [10, 11].

Consistent with its evolutionary significance as a psychological response to reduce harm exposure, PTSD has been documented in a range of cultures and harm contexts ranging from military service members in nation-state societies, subsistence pastoralists exposed to cattle raids [8, 12], child soldiers from various civil wars [13–15], refugees and those in postconflict zones [16–18] and victims of physical assault [19], rape [20], domestic violence [21] and motor vehicle accidents [22].

While cross-cultural work has aided in helping us parse the ultimate evolutionary causes of PTSD symptoms, similar work into the proximate physiological mechanisms of stress response is also needed. One suggestion is that PTSD manifests through an exacerbation of the acute stress response [23–28]. The acute stress response, commonly known as ‘fight–flight–freeze’, is activated by the sympathetic nervous system in response to stress [29]. When triggered by physiological or psychological stress, the acute stress response starts a cascade of hormonal signaling, increasing circulating cortisol. This cortisol momentarily suppresses functions not critical to immediate survival [30], increases available energy, and primes the immune response to physical harm [31]. However, if triggered inappropriately or at inappropriate times, the acute stress response could lead to dysregulation [32–34]. There is some evidence that adverse childhood environments can negatively alter adolescents’ baseline cortisol and stress responses [35–37].

Cortisol plays critical roles throughout the body, including immune and stress responses, regulation of glucose and protein homeostasis [36, 38, 39], and the regulation of the sleep–wake cycle [40]. Although it can be activated by physical activity or stressful events, cortisol levels typically follow a diurnal rhythm. They are elevated upon waking, increase for approximately 30 minutes, gradually fall throughout the remaining waking hours, and then rise again during sleep [40]. Cortisol dysregulation may occur after habitual or chronic activation of the stress response. Typically, this dysregulation takes the form of ‘diurnal cortisol blunting’. Cortisol blunting is thought to occur through frequent or chronic acute stress response activation, which results in desensitization to cortisol and a diminished rise and fall of cortisol levels throughout the day [28].

Due to the important role of cortisol in the body, disruption of the typical circadian hormonal pattern could potentially negatively affect physical and mental health. For example, cortisol dysregulation in adolescents has been associated with an increased risk of developing PTSD [26, 39, 41, 42]. Dysregulation of the stress response can result in negative mental and physical health outcomes [28, 32, 43, 44]. Cortisol dysregulation has also been linked to the development of PTSD and the prolonged expression of PTSD symptoms [33].

Nonetheless, the overall relationship between baseline cortisol levels and PTSD is mixed [33]. Early studies detected differences in cortisol levels and diurnal patterns in populations with PTSD. For example, Yehuda et al. [23] separated subjects into three groups: one group of Vietnam combat veterans diagnosed with PTSD (*n* = 15), one group of outpatients diagnosed with clinical depression (*n* = 14), and one group of controls with no family history of major psychiatric disorders (*n* = 15). They found that subjects with PTSD had significantly lower cortisol levels than the clinically depressed subjects and presented with a ‘less rhythmic, more chaotic pattern of cortisol release’. Additionally, Yehuda et al. [45] examined the effects of aging and PTSD on cortisol rhythm within a group of Holocaust survivors with and without a PTSD diagnosis. They found that aged Holocaust survivors with PTSD had blunted diurnal cortisol rhythms. However, these results do not always replicate. Eckart et al. [46] investigated the relationship between PTSD and reduced cortisol levels in 43 male refugees who survived the Rwandan genocide, 24 with a diagnosis of PTSD and 19 without a diagnosis. They found no difference in diurnal cortisol between the groups. Early meta-analysis of 37 studies concluded that the PTSD populations (*n* = 828) and non-PTSD populations (*n* = 800) had no significant differences in baseline cortisol levels between groups [47].

Meta-analysis of more recent studies also found mixed results in an association between diurnal cortisol and PTSD [48–50]. One meta-analysis [48] concluded that individuals with PTSD had lower basal morning cortisol than controls and cortisol concentration later in the day was not associated with PTSD, indicating that the time and consistency of cortisol sampling is important for detecting associations between diurnal cortisol and PTSD. Additionally, Sterina et al. [51] found that trauma exposure earlier in the day, nearer to the post-awakening cortisol peak, was associated with lower PTSD symptoms up to six months after exposure. They hypothesized that time of trauma may be a factor in PTSD symptom expression because it alters circadian rhythm via glucocorticoid sensitive circadian connected genes.

Testosterone, another key physiological moderator of energetic homeostasis and response to acute stressors [52], also follows a diurnal rhythm with an awakening response similar to cortisol [53, 54]. While blunting of testosterone diurnal rhythm is less well studied than cortisol, studies do report that fragmented sleep can modify baseline and diurnal testosterone [52]. Additionally, the link between testosterone and PTSD symptoms has been under-explored compared to links between cortisol and PTSD. To the best of our knowledge, there are no meta-analyses of PTSD and testosterone to date. While a few studies have examined PTSD-testosterone links, the sample sizes have been small, and the results mixed [55–57].

Mixed results across different studies may be due to differences in cortisol sampling method (e.g. saliva vs. plasma), study populations, lab practices, PTSD assessment methods, types and duration of trauma, participants’ sleep duration and time of awakening, time since the trauma, and the timing of cortisol sample collection [48–50]. Some differences between studies may be because the types of traumatic events experienced by participants vary across and within studies, ranging from traffic accidents to repeated combat deployments or being a holocaust survivor [58]. Some studies use single-sex, and others use mixed-sex groups. Moreover, the time since the event varies greatly within studies. Studies using hormonal analyses typically focus on a single hormone and use different sampling and lab methods, making cross-study comparisons difficult. Finally, most studies on PTSD and cortisol were conducted with Western, industrialized populations and, therefore, are unlikely to provide a comprehensive picture of the diverse behavioral, psychological, and physiological responses to trauma [12, 59–67].

We conducted a study examining PTSD among Turkana pastoralists living in a semi-arid area of Kenya near the border with South Sudan [68]. The Turkana practice mobile pastoralism in the semi-arid savanna by keeping cattle, camel, sheep, goats, and donkeys and periodically migrating with some of their livestock to access fresh pastures and water sources. Their livestock-based subsistence strategy leaves them open to being raided by neighboring groups for their livestock and incentivizes their raiding of other ethnic groups to gain additional livestock themselves. Firearms started being adopted by the Turkana and neighboring pastoral groups who occupy areas along the border between Kenya, Uganda, South Sudan, and Ethiopia starting in the 1970s [69]. This helped to sustain the practice of livestock raiding in the region where members of multiple pastoralist ethnic groups vie for limited dry-season pastures [70]. Therefore, Turkana herders utilizing the border areas are constantly at risk of experiencing potentially lethal combat. For our study population, near the border between the Turkana in Kenya and Toposa pastoralists in South Sudan, nearly half of adult male mortality is due to raids [71]. In the specific Turkana community from which the current study sample was drawn, three-out-of-four men report having killed a person on a raid, one in four has a visible bullet wound, and approximately 28% of men would qualify for a provisional diagnosis of PTSD [8, 72].

Working with the Turkana offers a unique opportunity to study associations between PTSD and hormonal dysregulation in a non-western, non-industrialized subsistence population. No single population can characterize the diverse conditions under which human populations lived for long periods of our evolutionary history. However, the lifestyle of the Turkana may more closely reflect the constraints and adaptive challenges that could have shaped human physiology than the lifestyle of urban industrialized societies. They live in a subsistence-based economy, are a natural fertility population, have limited access to health care, and periodically experience stressors such as droughts, epidemics, and endemic warfare that affect food availability, morbidity, and mortality. Furthermore, unlike studies that are confounded by heterogeneity in the events that created PTSD and the length of time since those events, nearly all Turkana men in this study community constantly live under the threat of being attacked and have experienced combat either in the context of defending their livestock or when participating in offensive raids or both. Compared to some previous studies, our study population is relatively homogeneous in terms of the source of their trauma, cultural backgrounds, gender, and method of recruitment.

## METHODOLOGY

### Population

The study was conducted in a remote part of Northern Kenya about 80 kilometers northeast of the village of Kakuma near the border with South Sudan in the Ngikwatela territorial section of the Turkana. The study participants were a convenience sample of *n* = 66 male Turkana pastoralists who had participated in at least one cattle raid. We retained saliva samples from 60 participants. Turkana pastoralists generally do not know their age in calendar years. However, calendar ages can be estimated from their ‘age-set’, a cohort of individuals born over an approximately 3- to 6-year period [8]. We conducted this study among men believed to be between the ages of 18 and 65.

Individuals sampled in this study practice intensive semi-arid pastoralism and have high levels of physical activity and danger exposure. They keep a mix of cattle, sheep, goats, camels, and donkeys and subsist by consuming milk, blood, and meat from their livestock, supplemented with agricultural products they acquire by trading their livestock in markets. They are physically active and may walk many kilometers in a single day as they move their herds through grazing routes and between water sources. Individuals routinely walk 75 km or more to the market towns to sell livestock and purchase supplies. Young men and women separate from the wet-season settlements during the dry season and migrate more than 100 km away to access pastures and water.

Additionally, when men go on raids, they may need to walk more than 100 km to reach the territory of neighboring non-Turkana pastoral groups and may be forced to retreat quickly back to Turkana territory if they are being pursued by their opponents. They may also be attacked by members of other pastoral groups as they herd, as raids can happen during the day at pastures, watering sites, during migration, or at night while asleep in their homesteads.

While we did not collect detailed raiding history and demographic information from the subjects in this study, we have such data from subjects recruited from the same study population [8], which included some of the same participants. In that sample, combat exposure was high. The median was three battle raids, one stealth raid, two defensive raids, and four ambushes by hostile forces. 72% of participants reported having killed an enemy in combat, and 26% had at least one visible bullet wound from enemy fire. In addition, they periodically experience stress from loss of livestock to raids, disease epidemics, and droughts. A substantial drought was occurring during the study period.

A more detailed description of the ethnographic and ecological context of the study is included in the preregistration archive at https://doi.org/10.17605/OSF.IO/K87Y9.

### Study methods

The study was conducted with four cohorts of participants, with each cohort participating over a different 2-day period. On the first day, participants were enrolled and assessed for PTSD severity using the fifth edition of the PTSD Checklist (PCL-5) [73] that was translated into Turkana as described by Zefferman and Mathew [8] and administered verbally by local Turkana research assistants. The responses of 21 participants resulted in high PTSD severity scores (PCL-5 ≥ 33), and the responses of 39 resulted in low PTSD severity scores (PCL-5 < 33). A PCL-5 score of 33 is the standard cutoff used for a provisional diagnosis of PTSD in the clinical literature [73].

After this initial assessment, they were asked to return to the study site in the evening. The study site was near a small center with a mobile clinic, water tank, church, and a few stalls selling small supplies like tobacco, sugar, tea, etc. It is close to a semi-permanent pastoral settlement and a place where people congregate for conversation and meetings. At the time of the study, an NGO was training the pastoral community in this area to manage a farm close to the center, which drew people to pass by the location. Additionally, the drought incentivized people to visit the water tank. In the evening, study participants were fed a locally prepared meal, provided with a 500 ml bottle of water, and trained on how to use the bottle to wash their mouths in accordance with saliva collection protocols. They were also told to refrain from chewing tobacco.

Participants spent the night at the research location, sleeping communally outside, a natural sleeping arrangement for Turkana men. At 5:30 a.m., participants were gently woken, asked to rinse their mouths with a fresh bottle of water, and provided a passive saliva sample. Saliva samples were labeled and immediately frozen in liquid nitrogen.

After the first sample, participants were served a morning meal, and the sampling procedure was repeated at 8:00 a.m. and 11:00 a.m.. Participants refrained from eating or chewing tobacco the hour before each sample was taken. After we collected the third and final sample, participants were compensated with a portion of maize and sugar. Participants stayed in the study area during the entire period and were monitored by research staff.

Since the liquid nitrogen tank could only hold samples from 60 participants, samples from six participants were discarded. Two participants’ samples were discarded because they received invasive medical treatment during the study. Another participant’s samples were discarded because he left the study area before contributing all three samples, though he did complete the study in a later cohort. Three participants’ samples were discarded at random from the participants with low symptom severity to make room for the samples of participants with high symptom severity.

The study preregistration archive at https://doi.org/10.17605/OSF.IO/K87Y9 describes the surveying, sampling, and collection methods in much greater detail.

### Lab methods

Saliva specimens were stored in liquid nitrogen for up to two months until transferred on dry ice from Kenya to Arizona State University. Once in the lab, specimens were stored at −80C for one week before cortisol analyses. Specimens were thawed and centrifuged for 15 minutes at 1500*g*, and the aqueous layer was aliquoted for immediate analysis. Three samples from the same individual had visual evidence of blood contamination and were removed from analyses, and four specimens had insufficient volume. All specimens from each individual were run on the same plate, and individuals were randomized across plates to ensure no batch effects. Cortisol and testosterone were measured via enzyme immunoassay (Cortisol PAb R4866, Testosterone PAb R156/7, both supplied by Coralie Munro), and specimens were diluted 1:2 in 0.1% BSA PBS [74]. The within and between plate coefficients of variation were 13.9% and 15.9% for the low control and 5.0% and 16.6% for the high control.

### Statistical analysis

The cortisol sensitivity analysis for this project was preregistered at https://doi.org/10.17605/OSF.IO/K87Y9, and the testosterone sensitivity analysis was preregistered at https://doi.org/10.17605/OSF.IO/VHA24. Deviations from the preregistered protocol are described below.

**Figure 1.**
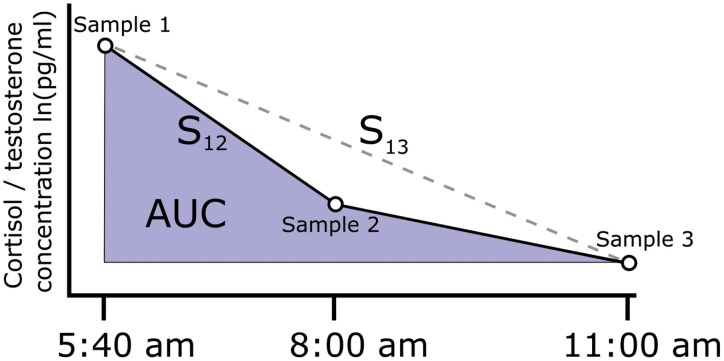
The three sensitivity measures we preregistered for this analysis using a typical diurnal curve from the literature. S_12_ is the slope of the biomarker concentration from the first to the second sample. S_13_ is the slope of the biomarker concentration from the first to the third sample. AUC is the area under the biomarker concentration curve.

Our preregistered analysis included three prominent measures of cortisol and testosterone sensitivity, as shown in Figure 1, as response variables. The first measure, S_12_, is the slope in the log change in cortisol or testosterone concentration from the first to the second sample for each participant. The second, S_13_, is the slope in cortisol or testosterone concentration from the first to the third sample. With a typical diurnal concentration curve, more negative S_12_ and S_13_ slopes indicate greater biomarker sensitivity. The third, AUC, is the area under the cortisol or testosterone concentration curves from the first to the third sample. Smaller areas indicate greater sensitivity for the typical diurnal curve.

Our preregistration assumed that participants would have typical diurnal curves, where both cortisol and testosterone concentrations tended to decrease from the second to third samples. However, as described in our results, the concentration of cortisol and testosterone tended to increase in this study between the second and third samples. The meaning of S_13_ and AUC measures was therefore unclear. We, therefore, only report analysis of the S_12_ measure in the main text, though include the results of the S_13_ and AUC analysis in the Appendices S5—S9 in compliance with our preregistration.

Our preregistered analysis described fitting 24 linear models to each response variable. Twelve were fixed effect models, and 12 included the study cohort as a random effect to account for any unmeasured differences between the four study cohorts. However, the Markov chains for the random effects models often fail to converge. Further analysis suggested that this failure was due to the disproportional number of high-symptom-severity participants in one cohort, likely making it difficult to distinguish cohort effects from PTSD symptom severity. We, therefore, only report the analysis of 12 fixed effect models in the main text. We include the causal diagrams for the random effect models in Appendix S3 and the results of the analysis including random effects in Appendices S4-S9, though with the caveat that the parameter estimates may be fragile due to failure to converge. The 12 fitted models and corresponding causal models are summarized in Table 2. The variables used in the models are described in Table 1.

**Table 1.** Variables for statistical and causal models fit in the main text.

Variables	Description
Total symptom severity score (T)	A numeric variable from 0 to 80. The score is calculated as the sum of a participant’s symptom severity scores for all twenty PTSD symptoms.
High-symptom severity (H)	An indicator variable where a one indicates a participant reported high combat stress severity (*T > *33), and 0 indicates that they reported low combat stress severity (*T ≤ *33). This cutoff is from the clinical literature (e.g. Weathers et al., 2013).
Provisional PTSD diagnosis (P)	An indicator variable where a 1 indicates that a participant has high combat stress severity, and a 0 indicates that they have low combat stress severity using the standard clinical diagnostic criteria for PTSD from the DSM-5, which involves having a certain number of symptoms with high severity in different categories (American Psychiatric Association, 2013).
Depressive symptom severity(D)	A numeric variable from 0 to 24, calculated as the sum of the severity scores from participants’ ‘depressive’ symptoms. As defined by Zefferman and Mathew (2021), these symptoms are low concentration, detachment/estrangement, irritability/aggression, loss of interest, negative beliefs, negative feelings, and emotional numbing.
Learning/responding symptom severity (L)	A numeric variable from 0 to 24, calculated as the sum of the severity scores from participants’ ‘learning and responding’ symptoms. As defined by Zefferman and Mathew (2021), these symptoms are hypervigilance, increased startle response, intrusive memories, nightmares, flashbacks, cued emotional distress and cued physical reactions.
Estimated age (A)	An estimate of a participant’s age in years based on the estimated midpoint of their age-set’s range.
Environment (E)	Unmeasured aspects of the environment, including combat exposure and social support.
Cortisol/testosterone sensitivity (S)	Measure of sensitivity to cortisol and testosterone (S_12_, S_13_, or AUC).

**Table 2.** Statistical models fit in the main text of this paper with a diagram of each model’s causal interpretations. TS, HS, and PD share a causal interpretation as T, H, and P are alternative measures for the same symptom severity construct.

Model name	Causal models	Statistical models
Intercept	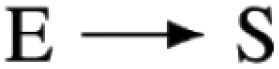	$S_{i}∼\beta_{0}$
Total severity (TS)	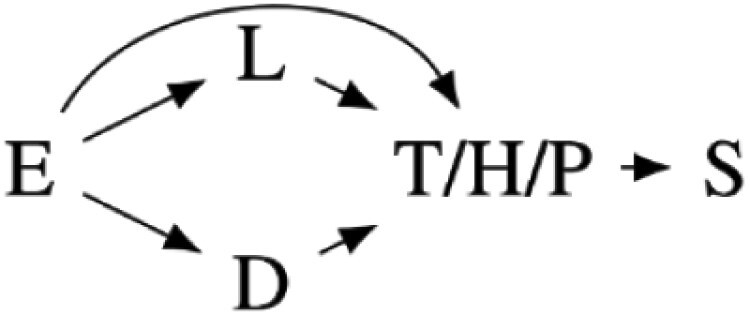	$S_{i}∼\beta_{0}+\;\beta_{T}T_{i}$
High severity (HS)		$S_{i}∼\beta_{0}+\;\beta_{H}H_{i}$
Provisional diagnosis (PD)		$S_{i}∼\beta_{0}+\;\beta_{P}P_{i}$
Depressive symptoms (DS)	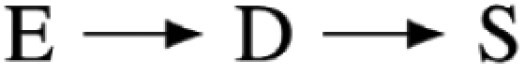	$S_{i}∼\beta_{0}+\;\beta_{D}D_{i}$
Learning & reacting severity (LRS)	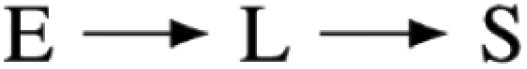	$S_{i}∼\beta_{0}+\;\beta_{L}L_{i}$
Age	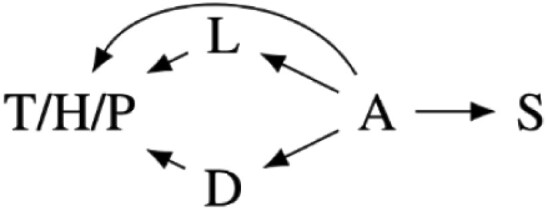	$S_{i}∼\beta_{0}+\;\beta_{\alpha }A_{i}$
Total severity + age (TS + age)	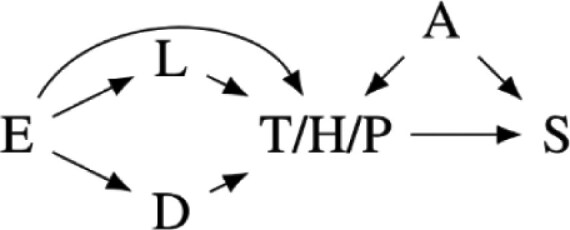	$S_{i}∼\beta_{0}+\;\beta_{T}T_{i}+\;\beta_{\alpha }A_{i}$
High severity + age (HS + age)		$S_{i}∼\beta_{0}+\;\beta_{H}H_{i}+\;\beta_{\alpha }A_{i}$
Provisional diagnosis + age (PD + age)		$S_{i}∼\beta_{0}+\;\beta_{P}P_{i}+\;\beta_{\alpha }A_{i}$
Depressive severity + age (DS + age)	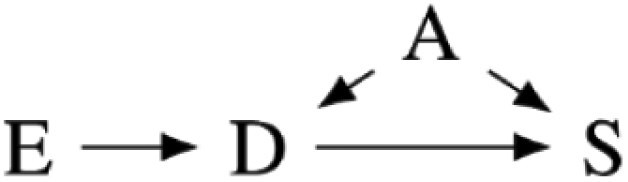	$S_{i}∼\beta_{0}+\;\beta_{D}D_{i}+\;\beta_{\alpha }A_{i}$
Learning & reacting severity + age (LRS + age)	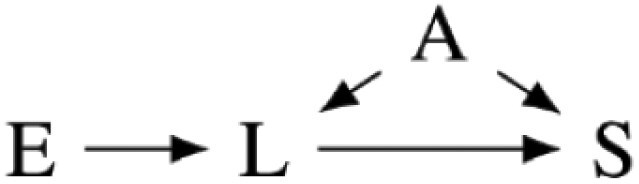	$S_{i}∼\beta_{0}+\;\beta_{LR}L_{i}+\;\beta_{\alpha }A_{i}$

We used three different measures of PTSD symptom severity. While actual PTSD diagnosis requires examination from a trained clinician, symptom severity can be assessed from a questionnaire. PTSD is a multidimensional clinical construct, the severity of which is typically assessed from a questionnaire in one of the three ways: an indicator variable for high-severity cases, an indicator variable for a provisional diagnosis of PTSD, or by treating the total severity score as a continuous variable. While there is a danger of dichotomizing complex variables [75] or using categorical variables as if they were continuous, we decided to fit models using all three measures of symptom severity as alternative predictors so that our results could be more easily compared with a variety of existing clinical literature.

Because a previous study of PTSD in this population [8] indicated that learning-and-reacting symptoms and depressive symptoms may be caused by distinct risk factors, we additionally looked at the relationship between cortisol and the severity of these specific symptom clusters. Previous studies showing the association of PTSD and cortisol have not separated these symptom clusters, so this analysis can shed light on whether the hormonal responses to PTSD are driven more by the learning-and-reacting symptoms or by the depressive symptoms.

We fit the regression models listed in Table 2 using the RStan package [76] for R. The full model specifications for the fitted models, including Bayesian priors, are in Appendices S1 and S2. The fitted models were compared with LOOIC weights calculated with the loo package [77–79]. We also conducted an exploratory analysis where each fixed effect model was fit using the lm() function in R, which calculated frequentist *P*-values for each parameter. Both procedures agreed on the same parameter values for each model.

### Ethics

The Arizona State University institutional review board approved this study. All participants provided informed consent.

## RESULTS

**Figure 2.**
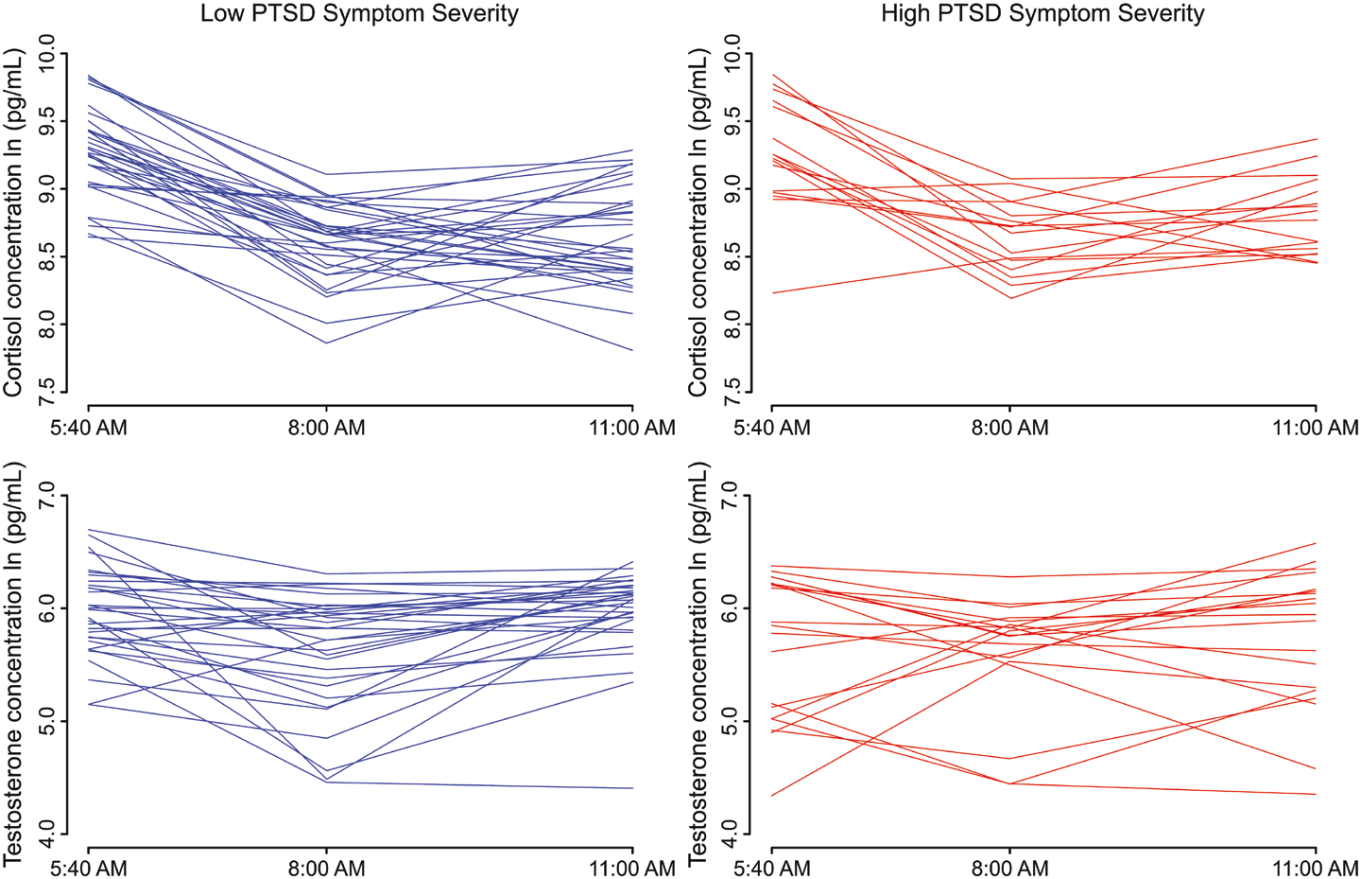
Change in concentrations of cortisol (top) and testosterone (bottom) for participants with low PTSD symptom severity (left) and high PTSD symptom severity (right). Contrary to our expectations, the concentration of both biomarkers tended to increase from the second to the third sample.

**Figure 3.**
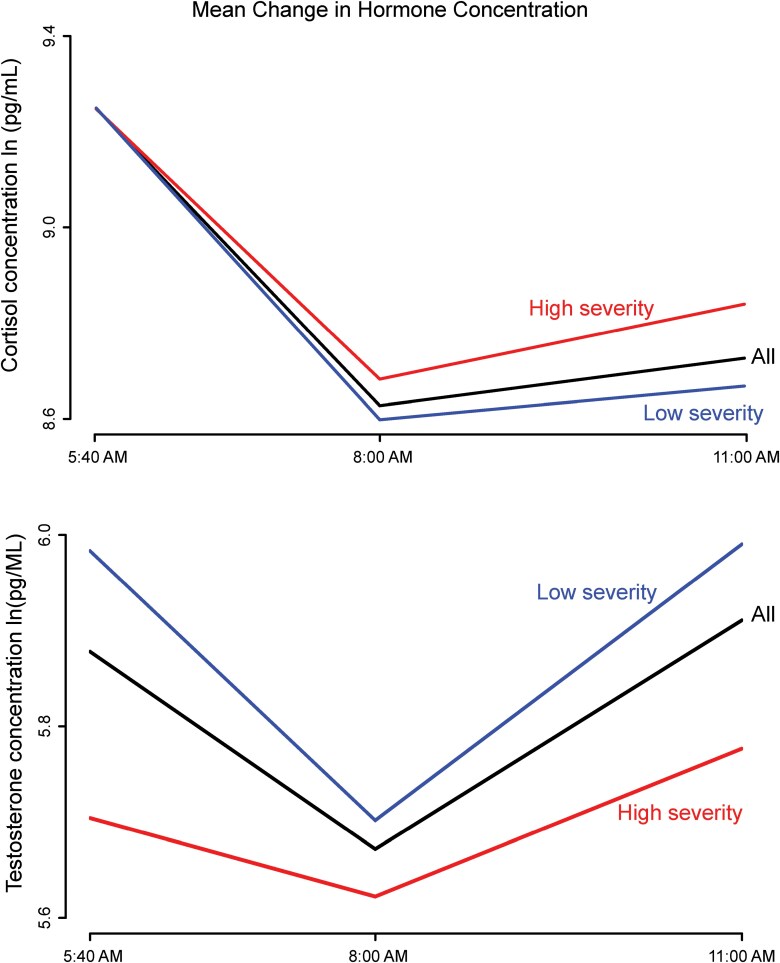
Mean concentration for measured cortisol (top) and testosterone (bottom) levels at each sample time for participants with high PTSD symptom severity (red), participants with low PTSD symptom severity (blue), and all participants (black). Concentrations of both hormones decrease, on average, between the first and second sample times and increase between the second and third.

Figure 2 shows the change in cortisol and testosterone concentrations for all participants over the three sample periods. Both cortisol and testosterone concentrations tended to be highest at the 5:40 a.m. sample and tended to decrease by the 8:00 a.m. sample. 93% of participants’ cortisol concentrations decreased from 5:40 a.m. to 8:00 a.m., and 77% had decreasing testosterone over the same period. However, contrary to the typical diurnal response curve in the literature, both cortisol and testosterone tended to increase by the 11:00 a.m. sample. 59% of participants had increasing cortisol, and 75% had increasing testosterone from 8:00 a.m. to 11:00 a.m.. Figure 3 shows that, on average, concentrations of both hormones decrease between the first two samples and increase in the third sample.

**Figure 4.**
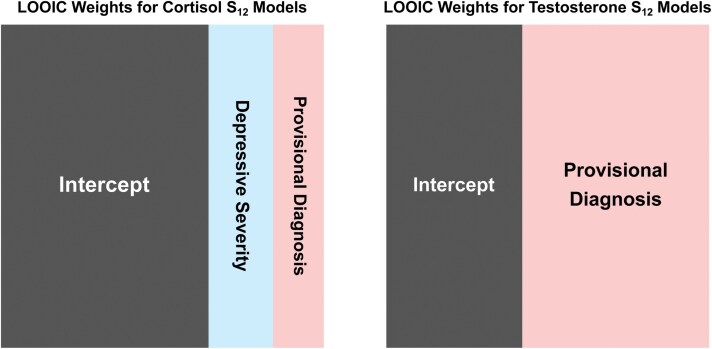
LOOIC model weights for the models with S_12_ (slope from T1 to T2) as the cortisol and testosterone sensitivity measures that are summarized in Tables 3 and 4. Areas represent the relative weight of the model. Three of the 12 models dominate the LOOIC Weights for cortisol sensitivity, with the most weight on the intercept model (with no predictor variables). For testosterone sensitivity, two of the 12 models dominate the LOOIC weights, with a model including the provisional diagnosis of PTSD as a predictor having the most weight and the intercept model having the second most weight. The models dominating the LOOIC Weight are not very distinguishable in terms of prediction, so there is not much evidence that any model performs better than the intercept model.

The parameter estimates and LOOIC weights for the fixed effects models fit to the cortisol S_12_ sensitivity measures are in Table 3 and the estimates and weights for the fixed effects models for the testosterone S_12_ sensitivity measure are reported in Table 4. Stars in both tables indicate standard statistical significance thresholds (i.e. one star indicates *P* < .05, and three stars indicate *P* < .001). LOOIC weights are also illustrated in Figure 4.

**Table 3.** Summary of linear models fit to the $S_{12}$ cortisol sensitivity measure as a response variable with parameter estimates and LOOIC estimates and weights. Slope coefficients indicate the average change in cortisol concentration, measured in ln(pg/mL), for a unit change in the variable adjusting for other variables in the model. Each variable’s units are described in Table 1.

Model name	$\beta_{0}$	$\beta_{TS}$	$\beta_{HS}$	$\beta_{PD}$	$\beta_{DS}$	$\beta_{LRS}$	$\beta_{age}$	σ	LOOIC	Weight
Intercept	−0.27***							0.17	−37.33	0.64
TS	−0.27***	0.000						0.17	−35.32	0.00
HS	−0.28***		0.037					0.17	−35.65	0.00
PD	−0.25***			−0.057				0.17	−36.36	0.16
DS	−0.29***				0.004			0.17	−36.34	0.20
LRS	−0.26***					−0.001		0.17	−35.44	0.00
Age	−0.28***						0.003	0.17	−35.41	0.00
TS + Age	−0.29***	0.000					0.003	0.17	−33.39	0.00
HS + Age	−0.29***		0.037				0.003	0.17	−33.67	0.00
PD + Age	−0.27***			−0.058			0.003	0.17	−34.53	0.00
DS + Age	−0.30***				0.004		0.001	0.17	−34.42	0.00
LRS + Age	−0.27***					−0.001	0.003	0.17	−33.47	0.00

**Table 4.** Summary of linear models fit to the $S_{12}$ testosterone sensitivity measure as a response variable with parameter estimates and LOOIC estimates and weights. Slope coefficients indicate the average change in testosterone concentration, measured in ln(pg/mL), for a unit change in the variable adjusting for other variables in the model. Each variable’s units are described in Table 1.

Model name	$\beta_{0}$	$\beta_{TS}$	$\beta_{HS}$	$\beta_{PD}$	$\beta_{DS}$	$\beta_{LRS}$	$\beta_{age}$	σ	LOOIC	Weight
Intercept	−0.10*							0.23	−2.03	0.42
TS	−0.14*	0.002						0.23	−0.73	0.00
HS	−0.14***		0.099					0.23	−2.81	0.00
PD	−0.14***			−0.158*				0.22	−5.52	0.58
DS	−0.13*				0.003			0.23	0.13	0.00
LRS	−0.15*					0.003		0.23	−1.00	0.00
Age	−0.11						0.002	0.24	−0.18	0.00
TS + age	−0.15	0.002					0.001	0.24	1.18	0.00
HS + age	−0.14		0.099				0.000	0.23	−0.83	0.00
PD + age	−0.14			0.160*			−0.002	0.23	−3.58	0.00
DS + age	−0.13				0.003		0.000	0.24	2.05	0.00
LRS + age	−0.15					0.003	0.001	0.24	0.91	0.00

We do not see strong evidence that PTSD symptom severity influences cortisol diurnal variation in this population. For the models that fit the cortisol S_12_ response variable, the Intercept model has the most LOOIC weight. For the models that fit the testosterone S_12_ response variable, the Intercept model had the second most LOOIC weight, closely behind the model using a Provisional Diagnosis of PTSD as a predictor, indicating that a provisional PTSD diagnosis does appear to be associated with lower waking testosterone, and less diurnal variation.

Although the Provisional Diagnosis models outperformed the Intercept model for the testosterone sensitivity models and had the second highest for the cortisol sensitivity models, they were very close to the Intercept models in expected predictive ability as measured by LOOIC weights. They also had opposite associations between the testosterone and cortisol models. For the cortisol sensitivity model, participants with a provisional diagnosis of PTSD had a more negative S_12_ value, indicating a more negative slope and, therefore, more cortisol sensitivity. This finding is contrary to the expectation from previous studies [23, 45–47]. For the testosterone sensitivity model, participants with a provisional diagnosis of PTSD had a more positive S_12_ value, indicating a less negative slope and, therefore, less testosterone sensitivity. This finding is in line with the expectations from previous studies but is opposite to the finding for the cortisol model.

Since it is also unclear why causally similar models to the Provisional Diagnosis model (i.e. those using Total Symptom Severity or High Severity as predictor variables) performed poorly, we do not think the evidence of a slight difference in LOOIC weights is strong enough to abandon the Intercept model. Furthermore, the parameter estimates for the hierarchical models reported in Appendices S4 and S7 do not change our overall conclusion.

**Figure 5.**
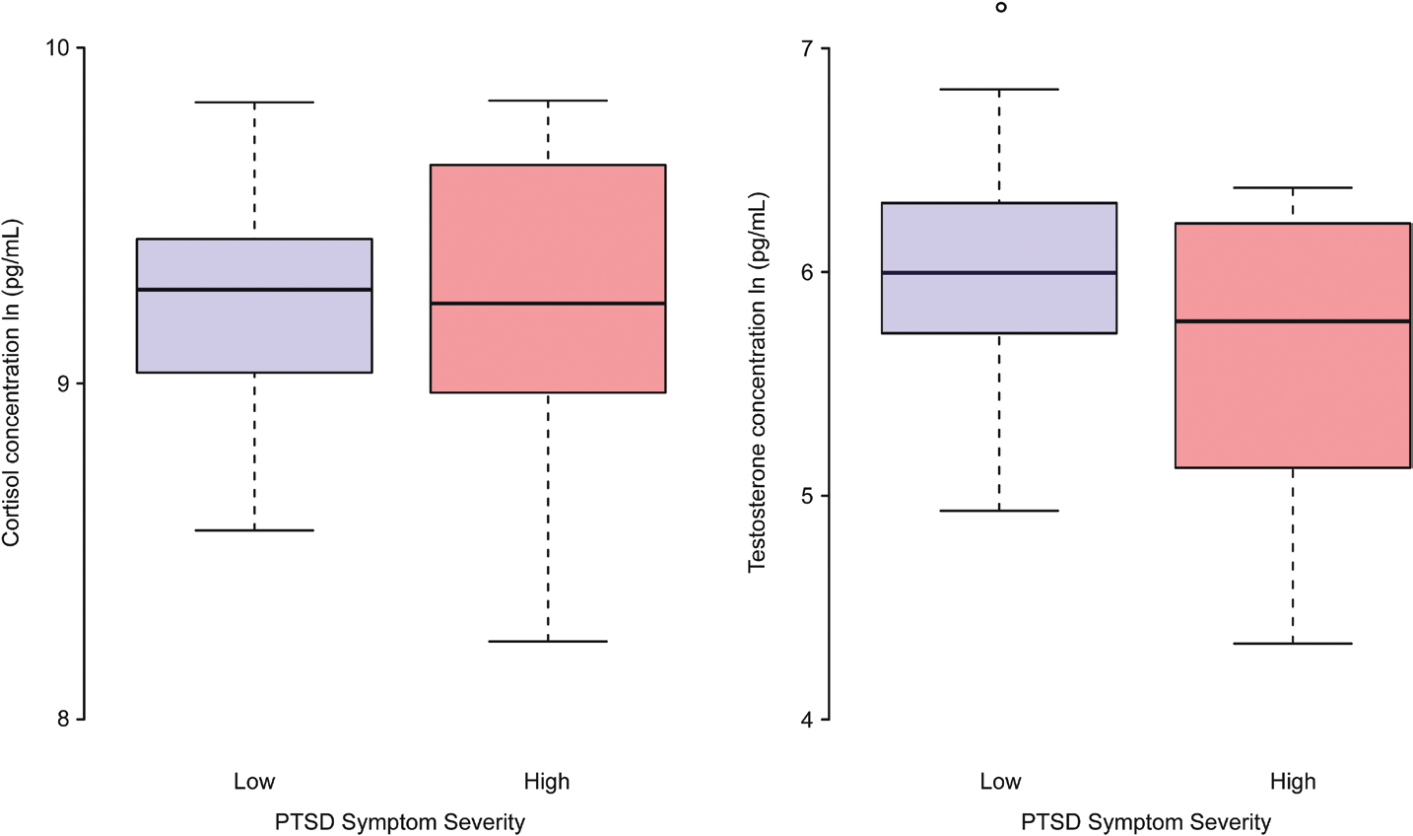
Waking cortisol and testosterone concentrations taken at waking for the population. The difference in waking cortisol concentration between low and high PTSD symptom severity participants was not significant. However, the waking testosterone concentration for the high PTSD severity group was significantly lower. This analysis was exploratory.

In an unregistered analysis, we compared the waking concentrations of cortisol and testosterone for participants with high and low PTSD symptom severity, as summarized in Figure 5. We also compared the waking concentrations between each group of participants with a standard two-tailed *t*-test. We found that the differences in waking cortisol concentrations were not significant (*P* = .99), but the waking testosterone concentration for the high PTSD severity group was significantly lower (*P* = .02). This exploratory analysis warrants further study.

## DISCUSSION

We found no clear association between PTSD and blunting of cortisol rhythms among adult Turkana men who have relatively high rates of combat exposure. We did find evidence that waking testosterone is lower in men with provisional PTSD diagnosis. Our results suggest that PTSD may not necessarily lead to blunted cortisol profiles and that the previously documented associations in Western industrialized study populations could stem from other correlated life factors among trauma survivors.

As we described earlier, Turkana men who herd and participate in raids have high levels of physical activity, as they will need to walk long distances over rough terrain almost daily. Studies in Western populations have found that higher levels of physical activity are associated with steeper cortisol slopes [80]. Their high levels of physical activity could be one reason why Turkana men, even those who exhibit PTSD symptoms, do not have blunted cortisol profiles. While waking cortisol was not associated with PTSD diagnosis, we did find that waking testosterone was lower in men with a provisional PTSD diagnosis.

Cultural factors can affect the acute stress response. In a seminal study, Cohen et al. showed that Americans who grew up in the South showed greater cortisol elevation when they were bumped into and insulted, than did Northerners in the same experimental condition [81]. Similarly, Park et al. show that negative affect was associated with a blunting in diurnal cortisol profile in American subjects, but not in Japanese subjects [82]. A key difference between the Turkana and nation-state societies is that Turkana warriors are strongly endorsed by their entire community, have elevated status, perform culturally sanctioned rituals after raids, and are deeply integrated with their corresponding civilian community [8]. These longstanding cultural traditions of warriorhood may buffer them from some of the negative physiological responses to combat trauma. Our findings highlight the importance of cross-cultural studies, especially with populations living outside of Western urbanized cultural contexts, to better understand how trauma exposure influences the acute stress response system.

While cross-cultural work has aided in our understanding of the ultimate evolutionary causes of war and associated combat stress symptoms [8, 72, 83], similar work into the proximate physiological mechanisms of stress response is also needed. This study is a step in that direction by avoiding many of the pitfalls of previous research by studying hormone-PTSD interactions in a non-industrial sample of individuals who have all experienced repeated warfare-related trauma. While many areas of evolutionary biology can use animal models to conduct causal studies (e.g. inducing obesity in murine models to study cardiovascular disease), the psychological mechanisms for mice are extremely different from humans, and our extreme sociality makes humans unique and highly divergent from even our closest living primate relatives [84]. More cross-cultural observational research like the current study is needed to better understand human variation and begin to parse the potential evolutionary implications of PTSD-hormone interactions.

## LIMITATIONS

One limitation of our study was that the size of our liquid nitrogen tank limited the number of saliva samples we could retain for each participant. For example, while a fourth saliva specimen for each participant would have allowed us to look at cortisol/testosterone awakening response, it would have also reduced our participant sample size by a quarter. When designing the study, we decided to have a larger participant sample size with fewer saliva samples each. This trade-off was in line with previous studies, which largely focused on variation across the day and not the awakening response [23, 28, 45].

While our study was conceived to examine if PTSD affects diurnal variability in cortisol and testosterone, it was not designed to examine other relationships between cortisol, testosterone, and PTSD symptoms. For example, individuals with and without PTSD may only differ in cortisol sensitivity during or immediately after exposure to acute stress. Some studies have experimentally examined this possibility in Western contexts by having participants dip their hands in an ice bucket [85]. Since we did not leverage natural stressors, such as raids, or experimental stressors, such as ice-bucket exposure, we cannot determine differences in cortisol sensitivity due to acute stress. Additionally, since the Turkana, unlike Western populations, showed an increase in cortisol concentration after an initial dip, their overall diurnal cortisol rhythm may be different from Western subjects. Therefore, a more detailed picture of population-specific hormonal variability across a 24-hour period may be needed to expose any differences in diurnal cortisol rhythm in the Turkana with high and low PTSD symptom severity.

Unlike in similar studies in the Western context, our participants slept together in a centralized location and had similar sleeping and waking times. This makes our results more challenging to compare with previous studies where participants slept in their domiciles and had different sleeping and waking times. We decided to have all participants spend the night in the same location to eliminate confounding variation. For example, some participants may have co-slept with their wives and children, while others would have slept alone. Previous studies across varied ecological contexts, including East African pastoralists, report that men who co-sleep with children have lower testosterone [86, 87].

Turkana pastoralists also spend many nights in the open with their herds and away from their family’s homestead, often around other men for socialization and protection from raids. Also, since sleep offsets have been found to have much less variability in subsistence populations that rise with the sun [88], we reasoned that waking participants just before sunrise would minimize variation in waking time while still maintaining ecological validity. Additionally, these trade-offs between minimizing confounding variation and ecological validity made sense for practical and safety reasons in that we would not have been able to track and collect samples from nomadic pastoralists spread across the landscape—often in places where our research team could not safely venture.

The effect of combat stress in this population may also have been masked by other stress factors. For example, at the time of sampling, the Turkana pastoralists in our study region were in the midst of a severe drought that killed several hundred thousand of the livestock on which they relied. Previous studies have shown that prolonged natural disasters in subsistence populations can result in low cortisol levels in multi-stressor environments [74]. While the mobile pastoral subsistence strategy of the Turkana is a cultural adaptation to the semi-arid savanna conditions where droughts periodically occur, and the Turkana may also have some drought-tolerant genetic adaptions [89], natural disasters like drought and accompanying hunger-related stressors can affect the stress response system. Thus, it is possible that the impacts of drought on cortisol or the acute stress response could have overshadowed any individual-level effects of PTSD on cortisol. Specifically, the impact of the drought and associated psycho-social stress and low food availability may have generally blunted all diurnal cortisol variation, which could have obfuscated any association between PTSD and cortisol. We do not have anthropometric data from before or after the drought, so we cannot assess individual-level changes in body fat or body mass before and after food insecurity. While no difference in cortisol levels has been previously reported between pastoralists versus urban settled Turkana [90], both herders as well as settled urban Turkana experience high food insecurity during droughts. It is expensive to maintain a high testosterone phenotype, and thus during major physiological stress, testosterone is often low [91–93], so it may be that the current environmental stressors overshadowed any impact of PTSD on testosterone.

## Supplementary Material

eoaf004_suppl_Supplementary_Materials

## Data Availability

Anonymized data and model code are available in an OSF archive at https://osf.io/bjdz5/ [94].
