# Supplementary material for: Little evidence that posttraumatic stress is associated with diurnal hormone dysregulation in Turkana pastoralists
Source: Evol Med Public Health. 2025 Feb 17;13(1):77–91. doi: 10.1093/emph/eoaf004 (PMC11973635; doi:10.1093/emph/eoaf004)
Supplement: eoaf004_suppl_Supplementary_Materials [file eoaf004_suppl_supplementary_materials.pdf]

# Supplementary materials: Little evidence that posttraumatic stress is associated with diurnal hormone dysregulation in Turkana pastoralists

## S1 Cortisol model specifications

These are the full model specifications for the fitted models described in Table 3 of the main text.

**Intercept:**

$$\beta_0 \sim \mathcal{N}(0,3)$$
$$\sigma \sim \mathcal{N}(0,3)$$
$$S_i \sim \mathcal{N}\left(\beta_0, \sigma\right)$$

**Total Severity (TS):**

$$\beta_0 \sim \mathcal{N}(0,3)$$
$$\beta_T \sim \mathcal{N}(0,1)$$
$$\sigma \sim \mathcal{N}(0,3)$$
$$S_i \sim \mathcal{N}\left(\beta_0 + \beta_T T_i, \sigma\right)$$

**High Severity (HS):**

$$\beta_0 \sim \mathcal{N}(0,3)$$
$$\beta_H \sim \mathcal{N}(0,1)$$
$$\sigma \sim \mathcal{N}(0,3)$$
$$S_i \sim \mathcal{N}\left(\beta_0 + \beta_H H_i, \sigma\right)$$

**Provisional Diagnosis (PD):**

$$\beta_0 \sim \mathcal{N}(0,3)$$
$$\beta_P \sim \mathcal{N}(0,1)$$
$$\sigma \sim \mathcal{N}(0,3)$$
$$S_i \sim \mathcal{N}\left(\beta_0 + \beta_P P_i, \sigma\right)$$

**Depressive Severity (DS):**

$$\beta_0 \sim \mathcal{N}(0,3)$$
$$\beta_D \sim \mathcal{N}(0,1)$$
$$\sigma \sim \mathcal{N}(0,3)$$
$$S_i \sim \mathcal{N}\left(\beta_0 + \beta_D D_i, \sigma\right)$$

**Learning & Reacting Severity (LRS):**

$$\beta_0 \sim \mathcal{N}(0, 3)$$
$$\beta_L \sim \mathcal{N}(0, 1)$$
$$\sigma \sim \mathcal{N}(0, 3)$$
$$S_i \sim \mathcal{N}\left(\beta_0 + \beta_L L_i, \sigma\right)$$

**Age:**

$$\beta_0 \sim \mathcal{N}(0, 100)$$
$$\beta_\alpha \sim \mathcal{N}(0, 20)$$
$$\sigma \sim \mathcal{N}(0, 3)$$
$$S_i \sim \mathcal{N}\left(\beta_0 + \beta_\alpha A_i, \sigma\right)$$

**Total Severity + Age (TS + Age):**

$$\beta_0 \sim \mathcal{N}(0, 3)$$
$$\beta_T \sim \mathcal{N}(0, 1)$$
$$\beta_\alpha \sim \mathcal{N}(0, 1)$$
$$\sigma \sim \mathcal{N}(0, 3)$$
$$S_i \sim \mathcal{N}\left(\beta_0 + \beta_T T_i + \beta_\alpha A_i, \sigma\right)$$

**High Severity + Age (HS + Age):**

$$S_i \sim \beta_0 + \beta_H H_i + \beta_\alpha A_i$$
$$\beta_0 \sim \mathcal{N}(0, 3)$$
$$\beta_H \sim \mathcal{N}(0, 1)$$
$$\beta_\alpha \sim \mathcal{N}(0, 1)$$
$$\sigma \sim \mathcal{N}(0, 3)$$
$$S_i \sim \mathcal{N}\left(\beta_0 + \beta_H H_i + \beta_\alpha A_i, \sigma\right)$$

**Provisional Diagnosis + Age (PD + Age):**

$$\beta_0 \sim \mathcal{N}(0, 3)$$
$$\beta_P \sim \mathcal{N}(0, 1)$$
$$\beta_\alpha \sim \mathcal{N}(0, 1)$$
$$\sigma \sim \mathcal{N}(0, 3)$$
$$S_i \sim \mathcal{N}\left(\beta_0 + \beta_P P_i + \beta_\alpha A_i, \sigma\right)$$

**Depressive Severity + Age (DS + Age):**

$$\beta_0 \sim \mathcal{N}(0, 3)$$

$$\beta_D \sim \mathcal{N}(0, 1)$$

$$\beta_\alpha \sim \mathcal{N}(0, 1)$$

$$\sigma \sim \mathcal{N}(0, 3)$$

$$S_i \sim \mathcal{N}\left(\beta_0 + \beta_D D_i + \beta_\alpha A_i, \sigma\right)$$

**Learning & Reacting Severity + Age (LRS + Age):**

$$\beta_0 \sim \mathcal{N}(0, 3)$$

$$\beta_{LR} \sim \mathcal{N}(0, 1)$$

$$\beta_\alpha \sim \mathcal{N}(0, 1)$$

$$\sigma \sim \mathcal{N}(0, 3)$$

$$S_i \sim \mathcal{N}\left(\beta_0 + \beta_{LR} L_i + \beta_\alpha A_i, \sigma\right)$$

## S2 Testosterone model specifications

These are the full model specifications for the fitted models described in Table 4 of the main text.

**Intercept:**

$$\beta_0 \sim \mathcal{N}(0, 3)$$
$$\sigma \sim \mathcal{N}(0, 3)$$
$$S_i \sim \mathcal{N}\left(\beta_0, \sigma\right)$$

**Total Severity (TS):**

$$\beta_0 \sim \mathcal{N}(0, 3)$$
$$\beta_T \sim \mathcal{N}(0, 1)$$
$$\sigma \sim \mathcal{N}(0, 3)$$
$$S_i \sim \mathcal{N}\left(\beta_0 + \beta_T T_i, \sigma\right)$$

**High Severity (HS):**

$$\beta_0 \sim \mathcal{N}(0, 3)$$
$$\beta_H \sim \mathcal{N}(0, 1)$$
$$\sigma \sim \mathcal{N}(0, 3)$$
$$S_i \sim \mathcal{N}\left(\beta_0 + \beta_H H_i, \sigma\right)$$

**Provisional Diagnosis (PD):**

$$\beta_0 \sim \mathcal{N}(0, 3)$$
$$\beta_P \sim \mathcal{N}(0, 1)$$
$$\sigma \sim \mathcal{N}(0, 3)$$
$$S_i \sim \mathcal{N}\left(\beta_0 + \beta_P P_i, \sigma\right)$$

**Depressive Severity (DS):**

$$\beta_0 \sim \mathcal{N}(0, 3)$$
$$\beta_D \sim \mathcal{N}(0, 1)$$
$$\sigma \sim \mathcal{N}(0, 3)$$
$$S_i \sim \mathcal{N}\left(\beta_0 + \beta_D D_i, \sigma\right)$$

**Learning & Reacting Severity (LRS):**

$$\beta_0 \sim \mathcal{N}(0, 3)$$
$$\beta_L \sim \mathcal{N}(0, 1)$$
$$\sigma \sim \mathcal{N}(0, 3)$$
$$S_i \sim \mathcal{N}\left(\beta_0 + \beta_L L_i, \sigma\right)$$

**Age:**

$$\beta_0 \sim \mathcal{N}(0, 3)$$
$$\beta_\alpha \sim \mathcal{N}(0, 1)$$
$$\sigma \sim \mathcal{N}(0, 3)$$
$$S_i \sim \mathcal{N}\left(\beta_0 + \beta_\alpha A_i, \sigma\right)$$

**Total Severity + Age (TS + Age):**

$$\beta_0 \sim \mathcal{N}(0, 3)$$
$$\beta_\alpha \sim \mathcal{N}(0, 1)$$
$$\beta_T \sim \mathcal{N}(0, 0.1)$$
$$\sigma \sim \mathcal{N}(0, 3)$$
$$S_i \sim \mathcal{N}\left(\beta_0 + \beta_T T_i + \beta_\alpha A_i, \sigma\right)$$

**High Severity + Age (HS + Age):**

$$\beta_0 \sim \mathcal{N}(0, 3)$$
$$\beta_\alpha \sim \mathcal{N}(0, 1)$$
$$\beta_H \sim \mathcal{N}(0, 1)$$
$$\sigma \sim \mathcal{N}(0, 3)$$
$$S_i \sim \mathcal{N}\left(\beta_0 + \beta_H H_i + \beta_\alpha A_i, \sigma\right)$$

**Provisional Diagnosis + Age (PD + Age):**

$$\beta_0 \sim \mathcal{N}(0, 3)$$
$$\beta_\alpha \sim \mathcal{N}(0, 1)$$
$$\beta_P \sim \mathcal{N}(0, 1)$$
$$\sigma \sim \mathcal{N}(0, 3)$$
$$S_i \sim \mathcal{N}\left(\beta_0 + \beta_P P_i + \beta_\alpha A_i, \sigma\right)$$

**Depressive Severity + Age (DS + Age):**

$$\beta_0 \sim \mathcal{N}(0, 3)$$
$$\beta_\alpha \sim \mathcal{N}(0, 1)$$

$$\beta_D \sim \mathcal{N}(0, 0.1)$$

$$\sigma \sim \mathcal{N}(0, 3)$$

$$S_i \sim \mathcal{N}\left(\beta_0 + \beta_D D_i + \beta_\alpha A_i, \sigma\right)$$

**Learning & Reacting Severity + Age (LRS + Age):**

$$\beta_0 \sim \mathcal{N}(0, 3)$$

$$\beta_\alpha \sim \mathcal{N}(0, 1)$$

$$\beta_{LR} \sim \mathcal{N}(0, 0.1)$$

$$\sigma \sim \mathcal{N}(0, 3)$$

$$S_i \sim \mathcal{N}\left(\beta_0 + \beta_{LR} L_i + \beta_\alpha A_i, \sigma\right)$$

## S3 All Models Including Cohort

| **Causal Model** | **Statistical Models** |
|---|---|
| 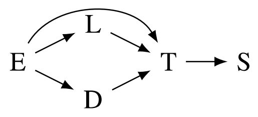 | $S_i \sim \beta_0 + \beta_T T_i$<br>$S_i \sim \beta_0 + \beta_T H_i$<br>$S_i \sim \beta_0 + \beta_P P_i$ |
| E → D → S | $S_i \sim \beta_0 + \beta_D D_i$ |
| E → L → S | $S_i \sim \beta_0 + \beta_L L_i$ |
| 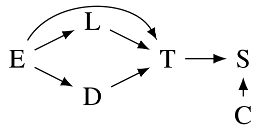 | $S_i \sim \beta_0 + \beta_T T_i + \beta_C C_i$<br>$S_i \sim \beta_0 + \beta_T H_i + \beta_C C_i$<br>$S_i \sim \beta_0 + \beta_P P_i + \beta_C C_i$ |
| 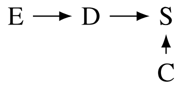 | $S_i \sim \beta_0 + \beta_D D_i + \beta_C C_i$ |
| 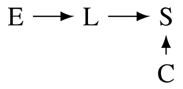 | $S_i \sim \beta_0 + \beta_{LR} L_i + \beta_C C_i$ |
| 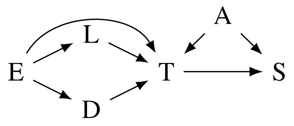 | $S_i \sim \beta_0 + \beta_T T_i + \beta_\alpha A_i$<br>$S_i \sim \beta_0 + \beta_T H_i + \beta_\alpha A_i$<br>$S_i \sim \beta_0 + \beta_P P_i + \beta_\alpha A_i$ |
| 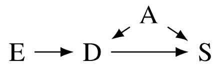 | $S_i \sim \beta_0 + \beta_D D_i + \beta_\alpha A_i$ |
| 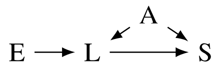 | $S_i \sim \beta_0 + \beta_{LR} L_i + \beta_\alpha A_i$ |
| 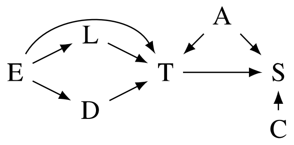 | $S_i \sim \beta_0 + \beta_T T_i + \beta_C C_i + \beta_\alpha A_i$<br>$S_i \sim \beta_0 + \beta_T H_i + \beta_C C_i + \beta_\alpha A_i$<br>$S_i \sim \beta_0 + \beta_P P_i + \beta_C C_i + \beta_\alpha A_i$ |
| 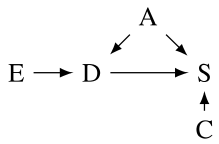 | $S_i \sim \beta_0 + \beta_D D_i + \beta_C C_i + \beta_\alpha A_i$ |
| 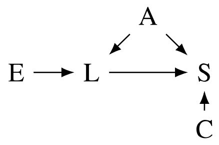 | $S_i \sim \beta_0 + \beta_{LR} L_i + \beta_C C_i + \beta_\alpha A_i$ |
| E → S | $S_i \sim \beta_0$ |
| C → S | $S_i \sim \beta_0 + \beta_C C_i$ |

| | |
|---|---|
| 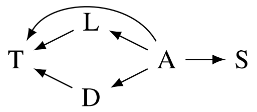 | $S_i \sim \beta_0 + \beta_\alpha A_i$ |
| 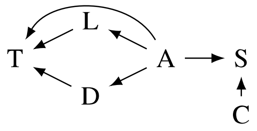 | $S_i \sim \beta_0 + \beta_C C_i + \beta_\alpha A_i$ |

Table S1: Causal and Statistical Models. For all models with cohort, $\beta_C \sim \mathcal{N}(\mu_j, \sigma^2)$.

## S4 S12 Cortisol Results With Cohort Random Effect

| **Model name** | $\beta_0$ | $\beta_{TS}$ | $\beta_{HS}$ | $\beta_{PD}$ | $\beta_{DS}$ | $\beta_{LRS}$ | $\beta_{age}$ | $\sigma_j$ | $\sigma$ | LOOIC | Weight |
|---|---|---|---|---|---|---|---|---|---|---|---|
| Intercept | -0.27 | | | | | | | | 0.17 | -37.30 | 0.33 |
| TS | -0.27 | 0.000 | | | | | | | 0.17 | -35.31 | 0.00 |
| HS | -0.28 | | 0.037 | | | | | | 0.17 | -35.63 | 0.00 |
| PD | -0.25 | | | -0.057 | | | | | 0.17 | -36.38 | 0.01 |
| DS | -0.29 | | | | 0.004 | | | | 0.17 | -36.35 | 0.10 |
| LRS | -0.26 | | | | | -0.001 | | | 0.17 | -35.42 | 0.00 |
| Age | -0.28 | | | | | | 0.003 | | 0.17 | -35.40 | 0.00 |
| TS + Age | -0.29 | 0.000 | | | | | 0.003 | | 0.17 | -33.36 | 0.00 |
| HS + Age | -0.29 | | 0.037 | | | | 0.003 | | 0.17 | -33.62 | 0.00 |
| PD + Age | -0.27 | | | -0.058 | | | 0.003 | | 0.17 | -34.51 | 0.00 |
| DS + Age | -0.30 | | | | 0.004 | | 0.001 | | 0.17 | -34.40 | 0.00 |
| LRS + Age | -0.27 | | | | | -0.001 | 0.003 | | 0.17 | -33.46 | 0.00 |
| Cohort | -0.26 | | | | | | | 0.11 | 0.16 | -35.40 | 0.27 |
| TS + Cohort | -0.26 | 0.000 | | | | | | 0.12 | 0.16 | -36.38 | 0.00 |
| HS + Cohort | -0.28 | | 0.041 | | | | | 0.12 | 0.16 | -36.84 | 0.00 |
| PD + Cohort | -0.28 | | | 0.041 | | | | 0.12 | 0.16 | -36.76 | 0.00 |
| DS + Cohort | -0.27 | | | | 0.020 | | | 0.12 | 0.16 | -37.90 | 0.13 |
| LRS + Cohort | -0.27 | | | | | 0.020 | | 0.12 | 0.16 | -37.93 | 0.15 |
| Age + Cohort | -0.29 | | | | | | 0.005 | 0.13 | 0.16 | -36.52 | 0.00 |
| TS + Age + Cohort | -0.29 | 0.000 | | | | | 0.005 | 0.13 | 0.16 | -34.40 | 0.00 |
| HS + Age + Cohort | -0.30 | | 0.038 | | | | 0.004 | 0.12 | 0.16 | -34.73 | 0.00 |
| PD + Age + Cohort | -0.28 | | | -0.054 | | | 0.006 | 0.12 | 0.16 | -35.32 | 0.01 |
| DS + Age + Cohort | -0.30 | | | | 0.003 | | 0.003 | 0.12 | 0.16 | -34.85 | 0.00 |
| LRS + Age + Cohort | -0.27 | | | | | -0.001 | 0.005 | 0.14 | 0.16 | -34.98 | 0.00 |

Table S2: Summary of linear models fit to the $S_{12}$ cortisol sensitivity measure as a response variable with parameter estimates and LOOIC estimates and weights including cohort as a random effect. Note that the models with cohort effects often failed to converge.

# S5 S13 Cortisol Results With Cohort Random Effect

| Model name | $\beta_0$ | $\beta_{TS}$ | $\beta_{HS}$ | $\beta_{PD}$ | $\beta_{DS}$ | $\beta_{LRS}$ | $\beta_{age}$ | $\sigma_j$ | $\sigma$ | LOOIC | Weight |
|---|---|---|---|---|---|---|---|---|---|---|---|
| Intercept | 47.1 | | | | | | | | 1.41 | 187.27 | 0.154 |
| TS | 46.7 | 0.014 | | | | | | | 1.40 | 187.70 | 0.113 |
| HS | 46.9 | | 0.525 | | | | | | 1.39 | 186.56 | 0.172 |
| PD | 47.0 | | | 0.465 | | | | | 1.40 | 187.22 | 0.114 |
| DS | 46.9 | | | | 0.033 | | | | 1.40 | 188.02 | 0.117 |
| LRS | 46.7 | | | | | 0.029 | | | 1.40 | 187.65 | 0.028 |
| Age | 46.9 | | | | | | 0.037 | | 1.42 | 189.58 | 0.063 |
| TS + Age | 46.6 | 0.013 | | | | | 0.030 | | 1.41 | 190.27 | 0.000 |
| HS + Age | 46.7 | | 0.511 | | | | 0.030 | | 1.40 | 189.10 | 0.001 |
| PD + Age | 46.8 | | | 0.440 | | | 0.026 | | 1.41 | 189.74 | 0.000 |
| DS + Age | 46.8 | | | | 0.031 | | 0.020 | | 1.42 | 190.71 | 0.000 |
| LRS + Age | 46.5 | | | | | 0.029 | 0.036 | | 1.41 | 190.15 | 0.000 |
| Cohort | 47.1 | | | | | | | 0.596 | 1.39 | 189.58 | 0.002 |
| TS + Cohort | 46.8 | 0.012 | | | | | | 0.415 | 1.39 | 188.60 | 0.014 |
| HS + Cohort | 46.9 | | 0.508 | | | | | 0.562 | 1.37 | 187.49 | 0.063 |
| PD + Cohort | 47.0 | | | 0.383 | | | | 0.518 | 1.39 | 188.78 | 0.000 |
| DS + Cohort | 47.1 | | | | 0.033 | | | 0.442 | 1.39 | 187.68 | 0.049 |
| LRS + Cohort | 47.1 | | | | | 0.032 | | 0.440 | 1.39 | 187.67 | 0.036 |
| Age + Cohort | 47.1 | | | | | | 0.005 | 0.590 | 1.39 | 24435.80 | 0.000 |
| TS + Age + Cohort | 46.8 | 0.012 | | | | | 0.003 | 0.542 | 1.39 | 815.24 | 0.000 |
| HS + Age + Cohort | 18.7 | | 0.543 | | | | 0.085 | 7.347 | 1.41 | 193.10 | 0.060 |
| PD + Age + Cohort | 18.7 | | | 0.216 | | | 0.098 | 7.358 | 1.43 | 194.51 | 0.014 |
| DS + Age + Cohort | 46.9 | | | | 0.026 | | -0.002 | 0.543 | 1.39 | 801.15 | 0.000 |
| LRS + Age + Cohort | 46.8 | | | | | 0.024 | 0.003 | 0.545 | 1.39 | 952.58 | 0.000 |

Table S3: Summary of linear models fit to the $S_{13}$ cortisol sensitivity measure as a response variable with parameter estimates and LOOIC estimates and weights including cohort as a random effect. Note that the models with cohort effects often failed to converge.

## S6 AUC Cortisol Results With Cohort Random Effect

| Model name | $\beta_0$ | $\beta_{TS}$ | $\beta_{HS}$ | $\beta_{PD}$ | $\beta_{DS}$ | $\beta_{LRS}$ | $\beta_{age}$ | $\sigma_j$ | $\sigma$ | LOOIC | Weight |
|---|---|---|---|---|---|---|---|---|---|---|---|
| Intercept | 47.1 | | | | | | | | 1.41 | 187.27 | 0.154 |
| TS | 46.7 | 0.014 | | | | | | | 1.40 | 187.70 | 0.113 |
| HS | 46.9 | | 0.525 | | | | | | 1.39 | 186.56 | 0.172 |
| PD | 47.0 | | | 0.465 | | | | | 1.40 | 187.22 | 0.114 |
| DS | 46.9 | | | | 0.033 | | | | 1.40 | 188.02 | 0.117 |
| LRS | 46.7 | | | | | 0.029 | | | 1.40 | 187.65 | 0.028 |
| Age | 46.9 | | | | | | 0.037 | | 1.42 | 189.58 | 0.063 |
| TS + Age | 46.6 | 0.013 | | | | | 0.030 | | 1.41 | 190.27 | 0.000 |
| HS + Age | 46.7 | | 0.511 | | | | 0.030 | | 1.40 | 189.10 | 0.001 |
| PD + Age | 46.8 | | | 0.440 | | | 0.026 | | 1.41 | 189.74 | 0.000 |
| DS + Age | 46.8 | | | | 0.031 | | 0.020 | | 1.42 | 190.71 | 0.000 |
| LRS + Age | 46.5 | | | | | 0.029 | 0.036 | | 1.41 | 190.15 | 0.000 |
| Cohort | 47.1 | | | | | | | 0.596 | 1.39 | 189.58 | 0.002 |
| TS + Cohort | 46.8 | 0.012 | | | | | | 0.415 | 1.39 | 188.60 | 0.014 |
| HS + Cohort | 46.9 | | 0.508 | | | | | 0.562 | 1.37 | 187.49 | 0.063 |
| PD + Cohort | 47.0 | | | 0.383 | | | | 0.518 | 1.39 | 188.78 | 0.000 |
| DS + Cohort | 47.1 | | | | 0.033 | | | 0.442 | 1.39 | 187.68 | 0.049 |
| LRS + Cohort | 47.1 | | | | | 0.032 | | 0.440 | 1.39 | 187.67 | 0.036 |
| Age + Cohort | 47.1 | | | | | | 0.005 | 0.590 | 1.39 | 24435.80 | 0.000 |
| TS + Age + Cohort | 46.8 | 0.012 | | | | | 0.003 | 0.542 | 1.39 | 815.24 | 0.000 |
| HS + Age + Cohort | 18.7 | | 0.543 | | | | 0.085 | 7.347 | 1.41 | 193.10 | 0.060 |
| PD + Age + Cohort | 18.7 | | | 0.216 | | | 0.098 | 7.358 | 1.43 | 194.51 | 0.014 |
| DS + Age + Cohort | 46.9 | | | | 0.026 | | -0.002 | 0.543 | 1.39 | 801.15 | 0.000 |
| LRS + Age + Cohort | 46.8 | | | | | 0.024 | 0.003 | 0.545 | 1.39 | 952.58 | 0.000 |

Table S4: Summary of linear models fit to the $AUC$ cortisol sensitivity measure as a response variable with parameter estimates and LOOIC estimates and weights including cohort as a random effect. Note that the models with cohort effects often failed to converge.

## S7 S12 Testosterone Results With Cohort Random Effect

| **Model name** | $\beta_0$ | $\beta_{TS}$ | $\beta_{HS}$ | $\beta_{PD}$ | $\beta_{DS}$ | $\beta_{LRS}$ | $\beta_{age}$ | $\sigma_j$ | $\sigma$ | LOOIC | Weight |
|---|---|---|---|---|---|---|---|---|---|---|---|
| Intercept | -0.10 | | | | | | | | 0.23 | -2.03 | 0.40 |
| TS | -0.14 | 0.002 | | | | | | | 0.23 | -0.73 | 0.00 |
| HS | -0.14 | | 0.099 | | | | | | 0.23 | -2.81 | 0.00 |
| PD | -0.14 | | | 0.158 | | | | | 0.22 | -5.52 | 0.20 |
| DS | -0.13 | | | | 0.003 | | | | 0.23 | 0.13 | 0.00 |
| LRS | -0.15 | | | | | 0.003 | | | 0.23 | -1.00 | 0.07 |
| Age | -0.11 | | | | | | 0.002 | | 0.24 | -0.18 | 0.00 |
| TS + Age | -0.15 | 0.002 | | | | | 0.001 | | 0.24 | 1.18 | 0.00 |
| HS + Age | -0.14 | | 0.099 | | | | 0.000 | | 0.23 | -0.83 | 0.00 |
| PD + Age | -0.14 | | | 0.159 | | | -0.002 | | 0.23 | -3.58 | 0.00 |
| DS + Age | -0.13 | | | | 0.003 | | 0.000 | | 0.24 | 2.05 | 0.00 |
| LRS + Age | -0.15 | | | | | 0.003 | 0.001 | | 0.24 | 0.91 | 0.00 |
| Cohort | -0.11 | | | | | | | 0.10 | 0.23 | -0.96 | 0.00 |
| TS + Cohort | -0.16 | 0.002 | | | | | | 0.11 | 0.23 | 0.46 | 0.00 |
| HS + Cohort | -0.16 | | 0.125 | | | | | 0.13 | 0.23 | -2.50 | 0.23 |
| PD + Cohort | -0.15 | | | 0.167 | | | | 0.11 | 0.22 | -4.31 | 0.10 |
| DS + Cohort | -0.13 | | | | 0.003 | | | 0.10 | 0.23 | 1.51 | 0.00 |
| LRS + Cohort | -0.16 | | | | | 0.004 | | 0.11 | 0.23 | 0.06 | 0.00 |
| Age + Cohort | -0.10 | | | | | | 0.000 | 0.09 | 0.24 | 0.84 | 0.00 |
| TS + Age + Cohort | -0.15 | 0.002 | | | | | -0.001 | 0.11 | 0.23 | 2.52 | 0.00 |
| HS + Age + Cohort | -0.14 | | 0.131 | | | | -0.003 | 0.14 | 0.23 | -0.27 | 0.00 |
| PD + Age + Cohort | -0.13 | | | 0.177 | | | -0.004 | 0.12 | 0.22 | -2.38 | 0.00 |
| DS + Age + Cohort | -0.12 | | | | 0.004 | | -0.002 | 0.11 | 0.24 | 3.87 | 0.00 |
| LRS + Age + Cohort | -0.16 | | | | | 0.004 | -0.001 | 0.11 | 0.23 | 2.02 | 0.00 |

Table S5: Summary of linear models fit the $S_{12}$ testosterone sensitivity measure as a response variable with parameter estimates and LOOIC estimates and weights, including cohort as a random effect. Note that the models with cohort effects often failed to converge.

# S8 S13 Testosterone Results With Cohort Random Effect

| **Model name** | $\beta_0$ | $\beta_{TS}$ | $\beta_{DS}$ | $\beta_{LRS}$ | $\beta_{HS}$ | $\beta_{PD}$ | $\beta_{age}$ | $\sigma_j$ | $\sigma$ | LOOIC | Weight |
|---|---|---|---|---|---|---|---|---|---|---|---|
| Intercept | 0.00 | | | | | | | | 0.11 | -84.69 | 0.10 |
| TS | 0.00 | 0.000 | | | | | | | 0.11 | -82.77 | 0.00 |
| HS | 0.00 | | 0.018 | | | | | | 0.11 | -82.62 | 0.00 |
| PD | -0.01 | | | 0.063 | | | | | 0.11 | -86.80 | 0.12 |
| DS | -0.02 | | | | 0.003 | | | | 0.11 | -85.09 | 0.11 |
| LRS | 0.01 | | | | | -0.001 | | | 0.11 | -82.71 | 0.00 |
| Age | -0.03 | | | | | | 0.005 | | 0.11 | -83.55 | 0.13 |
| TS + Age | -0.03 | 0.000 | | | | | 0.005 | | 0.11 | -81.62 | 0.00 |
| HS + Age | -0.03 | | 0.015 | | | | 0.005 | | 0.11 | -81.42 | 0.00 |
| PD + Age | -0.03 | | | 0.059 | | | 0.004 | | 0.11 | -85.19 | 0.04 |
| DS + Age | -0.04 | | | | 0.003 | | 0.003 | | 0.11 | -83.43 | 0.00 |
| LRS + Age | -0.02 | | | | | -0.001 | 0.005 | | 0.11 | -81.57 | 0.00 |
| Cohort | 0.00 | | | | | | | 0.06 | 0.11 | -85.13 | 0.22 |
| TS + Cohort | -0.01 | 0.000 | | | | | | 0.06 | 0.11 | -83.01 | 0.00 |
| HS + Cohort | -0.01 | | 0.024 | | | | | 0.07 | 0.11 | -82.83 | 0.00 |
| PD + Cohort | -0.02 | | | 0.063 | | | | 0.06 | 0.10 | -86.78 | 0.24 |
| DS + Cohort | -0.02 | | | | 0.003 | | | 0.06 | 0.10 | -85.60 | 0.05 |
| LRS + Cohort | 0.01 | | | | | -0.001 | | 0.07 | 0.11 | -82.78 | 0.00 |
| Age + Cohort | -0.03 | | | | | | 0.005 | 0.06 | 0.11 | -83.92 | 0.00 |
| TS + Age + Cohort | -0.03 | 0.000 | | | | | 0.005 | 0.06 | 0.11 | -81.67 | 0.00 |
| HS + Age + Cohort | -0.03 | | 0.019 | | | | 0.004 | 0.07 | 0.11 | -81.29 | 0.00 |
| PD + Age + Cohort | -0.03 | | | 0.059 | | | 0.003 | 0.06 | 0.10 | -84.88 | 0.00 |
| DS + Age + Cohort | -0.04 | | | | 0.003 | | 0.003 | 0.06 | 0.11 | -83.72 | 0.00 |
| LRS + Age + Cohort | -0.01 | | | | | -0.001 | 0.005 | 0.06 | 0.11 | -81.69 | 0.00 |

Table S6: Summary of linear models fit to the $S_{13}$ testosterone sensitivity measure as a response variable with parameter estimates and LOOIC estimates and weights including cohort as a random effect. Note that the models with cohort effects often failed to converge.

## S9 AUC Testosterone Results With Cohort Random Effect

| Model name | $\beta_0$ | $\beta_{TS}$ | $\beta_{HS}$ | $\beta_{PD}$ | $\beta_{DS}$ | $\beta_{LRS}$ | $\beta_{age}$ | $\sigma_j$ | $\sigma$ | LOOIC | Weight |
|---|---|---|---|---|---|---|---|---|---|---|---|
| Intercept | 30.8 | | | | | | | | 2.44 | 245.92 | 0.00 |
| TS | 31.7 | -0.034 | | | | | | | 2.39 | 244.03 | 0.13 |
| HS | 31.1 | | -0.727 | | | | | | 2.41 | 245.01 | 0.00 |
| PD | 31.0 | | | -0.427 | | | | | 2.44 | 246.55 | 0.00 |
| DS | 31.6 | | | | -0.103 | | | | 2.36 | 242.99 | 0.20 |
| LRS | 31.4 | | | | | -0.044 | | | 2.44 | 246.38 | 0.00 |
| Age | 32.9 | | | | | | -0.370 | | 2.19 | 236.07 | 0.36 |
| TS + Age | 33.5 | -0.028 | | | | | -0.353 | | 2.15 | 234.70 | 0.17 |
| HS + Age | 33.1 | | -0.633 | | | | -0.361 | | 2.17 | 235.60 | 0.10 |
| PD + Age | 32.9 | | | -0.183 | | | -0.365 | | 2.21 | 237.39 | 0.00 |
| DS + Age | 33.2 | | | | -0.068 | | -0.331 | | 2.16 | 235.57 | 0.00 |
| LRS + Age | 33.4 | | | | | -0.040 | -0.367 | | 2.19 | 236.45 | 0.00 |
| Cohort | 30.9 | | | | | | | 0.65 | 2.44 | 236.07 | 0.00 |
| TS + Cohort | 32.0 | -0.039 | | | | | | 0.57 | 2.36 | 245.01 | 0.00 |
| HS + Cohort | 31.2 | | -0.855 | | | | | 0.73 | 2.39 | 246.31 | 0.00 |
| PD + Cohort | 31.1 | | | -0.544 | | | | 0.72 | 2.43 | 248.19 | 0.00 |
| DS + Cohort | 31.5 | | | | -0.090 | | | 0.44 | 2.34 | 243.60 | 0.00 |
| LRS + Cohort | 31.5 | | | | | -0.046 | | 0.50 | 2.43 | 247.47 | 0.00 |
| Age + Cohort | 32.9 | | | | | | -0.382 | 0.63 | 2.19 | 237.82 | 0.00 |
| TS + Age + Cohort | 33.8 | -0.032 | | | | | -0.363 | 0.70 | 2.14 | 236.53 | 0.03 |
| HS + Age + Cohort | 33.1 | | -0.662 | | | | -0.367 | 0.65 | 2.17 | 237.44 | 0.00 |
| PD + Age + Cohort | 33.0 | | | -0.218 | | | -0.373 | 0.67 | 2.20 | 239.17 | 0.00 |
| DS + Age + Cohort | 33.3 | | | | -0.070 | | -0.345 | 0.63 | 2.16 | 237.69 | 0.00 |
| LRS + Age + Cohort | 33.6 | | | | | -0.045 | -0.384 | 0.66 | 2.19 | 238.30 | 0.00 |

Table S7: Summary of linear models fit to the $AUC$ testosterone sensitivity measure as a response variable with parameter estimates and LOOIC estimates and weights including cohort as a random effect. Note that the models with cohort effects often failed to converge.
